# Diagnostic value of repeated ice tests in the evaluation of ptosis in myasthenia gravis

**DOI:** 10.1371/journal.pone.0177078

**Published:** 2017-05-31

**Authors:** Jun Young Park, Hee Kyung Yang, Jeong-Min Hwang

**Affiliations:** Department of Ophthalmology, Seoul National University College of Medicine, Seoul National University Bundang Hospital, Seongnam, Gyeonggi-do, Korea; University of Sydney, AUSTRALIA

## Abstract

Twenty-six patients with ptosis related to Myasthenia gravis (MG) and 38 controls with ptosis other than MG were included. All patients were tested with the ice test 2 times on separate days in the afternoon. The margin reflex distance (MRD) was measured before and immediately after 2-minute application of ice on the eyelids. The ice test was judged positive if there was an improvement of at least 2.0 mm of MRD after the ice test. Among the patients with negative test results, 'equivocal’ was defined by improvement of MRD from at least 1.0 mm to less than 2.0 mm after the ice test. Repeated ice test results showed an agreement of 61.5% in MG, and 97.4% in nonmyasthenic ptosis. Repeated ice tests increased the sensitivity by 34.6% compared to a single test. Among the patients with repeatedly negative test results, 63.6% of those who showed equivocal results at least once turned out to be MG. Of those with repeated non-equivocal negative results, nobody turned out to be MG. There was no significant difference of the ice test results between ocular MG and generalized MG (*p* = 0.562).

## Introduction

Myasthenia gravis (MG) is a neuromuscular disorder caused by impaired synaptic transmission across the neuromuscular junction owing to acquired autoimmunity to the motor endplate, resulting in decreased available acetylcholine receptors (AChRs) [1]. Half of myasthenic patients initially present with ocular involvement [2]. Patients with MG commonly have ophthalmologic symptoms and signs including ptosis, diplopia, and ophthalmoplegia [3,4]. These manifestations may be variable or even absent depending on the time of examination. Diagnostic tests for MG can be performed in the office including the ice test [3], sleep test [5], rest test [6], anti-AChR antibody assay [7], Jolly test (electromyography with repetitive nerve stimulation) [8], neostigmine test [9,10] and antimyasthenic regimen trial [10]. However, none of these tests are 100% sensitive or specific [11]. Previous studies have reported that the ice test has a sensitivity of 90–95% and a specificity of 100% [3,6,12]. The major advantages of the ice test are its simple and noninvasive nature, short test duration and that no specialized equipment or trained personnel are required [13]. However, little is known of the repeatability of the ice test in myasthenic ptosis and controls. Herein, we report the results of the repeatability of the ice test in the evaluation of ptosis in MG.

## Materials and methods

### Participants

A retrospective observational study was conducted of 26 patients with ptosis related to MG and 38 controls with ptosis other than MG. All patients had at least 2 mm of ptosis and were studied between January 2012 and February 2014 at the neuro-ophthalmology clinic of Seoul National University Bundang Hospital (SNUBH). Patients with MG were diagnosed by an obvious history of diurnal variation of ptosis and/or fatigue and a positive result of at least one of the followings; AchR-Ab assay, neostigmine test, Jolly test, antimyasthenic regimen trial [10]. As for the control group, 38 patients with ptosis due to involutional blepharoptosis, oculomotor nerve palsy, thyroid associated ophthalmopathy, Miller-Fisher syndrome, congenital blepharoptosis, Horner’s syndrome and chronic progressive external ophthalmoplegia were included. This study was approved as a retrospective observational study by the Institutional Review Board (IRB) of Seoul National University Bundang Hospital before data collection (IRB No. B-1504-296-112), and adheres to the tenets of the Declaration of Helsinki. An informed consent from participants was not necessary since the ice test was examined for the purpose of evaluating patients with blepharoptosis.

### Ice test

**Fig 1:**
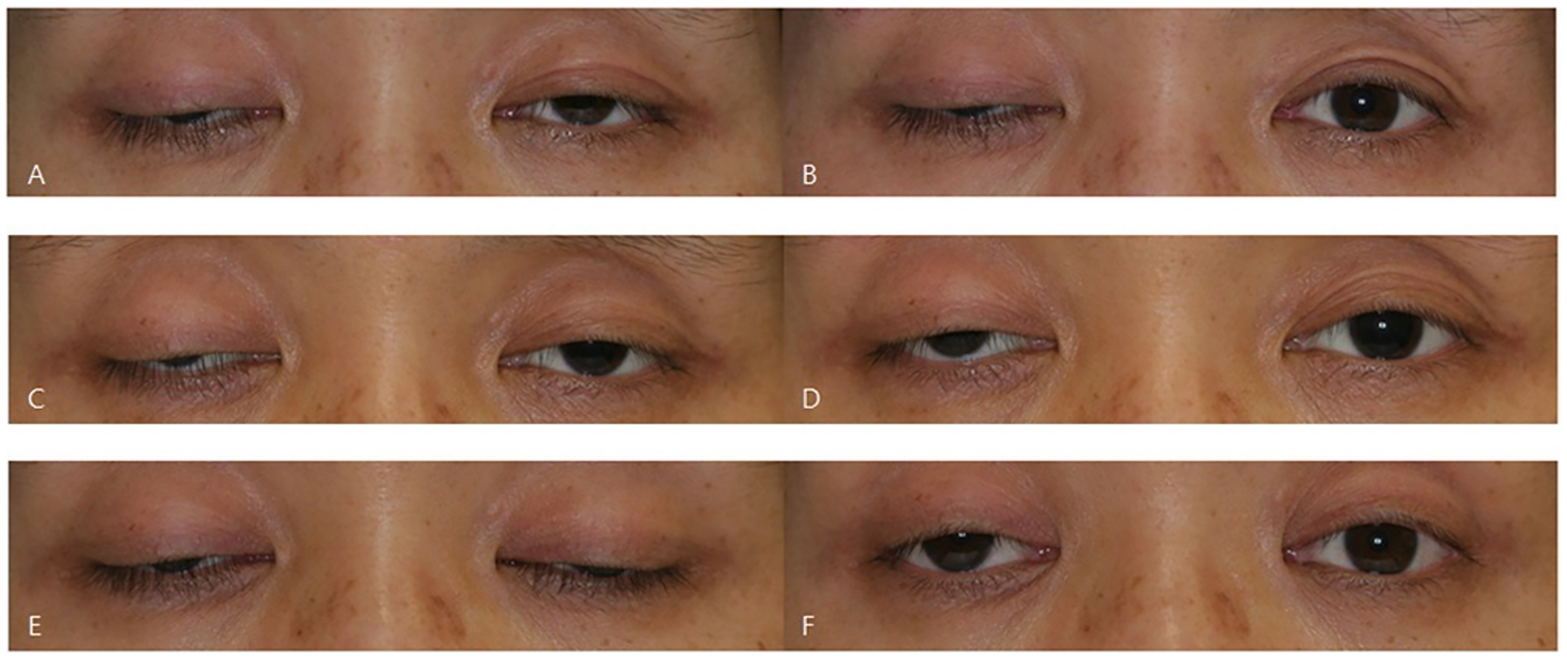
Variability of the ice test in myasthenia gravis. A patient with bilateral ptosis in myasthenia gravis, showing inconsistent results of the ice test performed on separate days in the right eye. (A) Asymmetric ptosis was present in both eyes prior to the ice test. (B) Immediately after 2-minute application of ice, ptosis in the left eye significantly improved with a fissure change of 2.5 mm. In contrast, the right eye showed a negative result. (C) Bilateral ptosis in the same patient on a different day prior to the ice test. (D) After the ice test, ptosis in the right eye improved with a fissure change of 1.0 mm, showing an equivocal negative response. (E) Bilateral ptosis in the same patient on a different day prior to the ice test. (F) After the test, ptosis improved with a fissure change of 2.0 mm in the right eye, showing a positive result.

All patients were tested with the ice test 2 times on separate days in the afternoon. The margin reflex distance (MRD) was measured before and immediately after 2-minute application of ice on the eyelids. MRD was defined as the distance between the center of the pupillary light reflex and the upper eyelid margin during primary gaze. All measurements were made with a millimeter ruler to the nearest 0.5 mm. The ice test was judged positive if there was an improvement of at least 2.0 mm MRD after the ice test (Fig 1) [14]. In case of a negative test result, ‘equivocal’ was defined by improvement of MRD from at least 1.0 mm to less than 2.0 mm after the ice test. After repeated ice tests, the maximum change in fissure size was noted. In cases of bilateral blepharoptosis, the data of only one eye with the larger change in fissure size after the ice test was included.

### Main outcome measures

The primary outcome was the repeatability of the ice test performed on separate days. Repeatability of the ice test was defined as the agreement (%) of repeated measurements in the same patient, which was either positive or negative, determined by a single examiner. Repeatability of change in fissure size after the ice test was calculated using intra-class correlation (ICC). The diagnostic value of the ice test including sensitivity, and specificity were also compared between repeated tests. Ice test results were compared using the independent t-test, χ^2^ test or Fisher’s exact test (SPSS software, version 15, SPSS, Inc.). A *p* value less than 0.05 was considered significant for all statistical tests.

## Results

### Patient demographics and baseline characteristics

In the MG group, there were 14 (53.8%) women and 12 (46.2%) men. Ages ranged from 3 to 77 years (mean 39.5 ± 23.5). The pre-test MRD was 0.3 ± 1.3 mm (range, -2.0~1.5) in MG patients.

In the control group, there were 18 (47.4%) women and 20 (52.6%) men without MG. Ages ranged from 3 to 75 years (mean 35.2 ± 25.1). The pre-test MRD was 0.8 ± 1.2 mm (range, -2.5~1.5) in controls (Table 1, S1 File).

**Table 1:** Demographics of patients with ptosis related to myasthenia gravis and controls.

	Myasthenia gravis	Control	p value
Number	26	38	
Age(yrs)	39.5 ± 23.5	35.2 ± 25.1	0.510^a^
Female gender	14 (53.8%)	18 (47.4%)	0.611^b^
Anti-AchR Antibody titer			
Positive	10 (38.5%)		
Negative	16 (61.5%)		
Jolly test			
Positive	9 (34.6%)		
Negative	17 (65.4%)		
Neostigmine test			
Positive	5 (19.2%)		
Negative	13 (50.0%)		
Not tested	8 (30.8%)		
Treatment			
Pyr	11 (42.3%)		
Pyr/Pred	8 (30.8%)		
AZP	1 (3.8%)		
AZP/Pred	1 (3.8%)		
Pre-test MRD (mm)	0.3 ± 1.3 (-2.0~1.5)	0.8 ± 1.2 (-2.5~1.5)	< 0.001^a^
Change in fissure size (mm)	1.9 ± 1.3 (0~4.0)	0.2 ± 0.8 (-1.5~3.0)	< 0.001^a^

Means ± SD (range), AChR = Acetylcholine receptor, Pyr = Pyridostigmine Bromide, Pred = Prednisolone, AZP = Azathioprine,

^a^Independent t-test,

^b^χ^2^ test

### Repeatability of the ice test

Repeated ice tests showed an agreement in 61.5% (16 of 26) of patients with MG, and 97.4% (37 of 38) of controls. Change in fissure size after the ice test showed poor repeatability in the MG group (ICC = 0.110, ranging from -0.985 to 0.601, *p* = 0.387), and fair repeatability in controls (ICC = 0.766, ranging from 0.550 to 0.879, *p* < 0.001).

### Diagnostic value of the ice test

On the first ice test, the positive predictive value (PPV) was 90.9% (10 of 11), negative predictive value (NPV) was 69.8% (37 of 53), sensitivity was 38.5% (10 of 26), and specificity was 97.4% (37 of 38) (Table 2). On the second ice test, the PPV was 93.3% (14 of 15), NPV was 75.5% (37 of 49), sensitivity was 53.8% (14 of 26), and specificity was 97.4% (37 of 38) (Table 3).

**Table 2:** The results of the first ice test.

Ice test^a^	Diagnosis		Total
	MG	Control	
Positive	10	1	11
Negative	16	37	53
Equivocal	13	2	15
Non-equivocal	3	35	38
Total	26	38	64

MG = Myasthenia gravis

^a^The ice test was positive if there was an improvement of at least 2.0 mm in MRD after the ice test. In case of a negative test result, equivocal was defined as an improvement in MRD of at least 1.0 mm to less than 2.0 mm after the ice test.

**Table 3:** The results of the second ice test.

Ice test^a^	Diagnosis		Total
	MG	Control	
Positive	14	1	15
Negative	12	37	49
Equivocal	6	4	10
Non-equivocal	6	33	39
Total	26	38	64

MG = Myasthenia gravis

^a^The ice test was positive if there was an improvement of at least 2.0 mm in MRD after the ice test. In case of a negative test result, equivocal was defined as an improvement in MRD of at least 1.0 mm to less than 2.0 mm after the ice test.

After repeated ice tests, 19 of 26 (73.1%) patients with MG and 1 of 38 patients (2.6%) without MG showed a positive test result at least once (*p* < 0.001, Fisher’s exact test). The PPV was 95.0% (19 of 20), NPV was 84.1% (37 of 44), sensitivity was 73.1% (19 of 26), and specificity was 97.4% (37 of 38) (Table 4). Repeated ice tests increased the PPV by 4.1%, NPV by 14.3% and sensitivity by 34.6% compared to the first ice test. Among the patients with repeatedly negative test results, 63.6% (7 of 11) of those who showed equivocal results at least once turned out to be MG. Of those with repeated non-equivocal negative results, none (0 of 33) turned out to be MG.

**Table 4:** The results of repeated ice tests.

Ice test^a^	Diagnosis		Total
	MG	Control	
Positive	19	1	20
Negative	7	37	44
Equivocal	7	4	11
Non-equivocal	0	33	33
Total	26	38	64

MG = Myasthenia gravis

^a^Repeated ice test results were considered positive if there was a positive test result at least once. When both test results were negative, it was judged as equivocal if there was an equivocal result at least once.

MG patients were subgrouped into ocular MG and generalized MG according to their association with systemic manifestations. However, there was no statistically significant difference in the results of the ice test between the two subgroups (*p* = 0.562, χ^2^ test).

The variability of MRD before application of the ice test (pre-test MRD) was determined. The mean variability of the pre-test MRD was 0.56 ± 0.53 mm (range, 0~2.0) in patients with MG and 0.25 ± 0.35 mm (range, 0~1.0) in controls, which was significantly larger in patients with MG (*p* < 0.001, independent t-test). After the ice test, there were no side effects except for mild local discomfort of the eyelids in all patients.

## Discussion

This is the first study reporting the repeatability of the ice test in the evaluation of ptosis in myasthenia gravis. Furthermore, there is a significant difference between our results and previous studies in regard of the 1) diagnostic value of the ice test and 2) interpretation of ‘equivocal’ results of the ice test.

A majority of patients with MG have a history of diurnal variation of ptosis and/or fatigue. Ocular manifestations in MG may be variable, from severe ptosis and/or diplopia to the point of being absent during an isolated examination [15]. Because of such characteristics, the ice test demonstrated poor repeatability in MG (61.5%) compared to nonmyasthenic ptosis (97.4%) in our study. As the symptoms of MG usually aggravate during the evening, we had all patients take the test in the afternoon. Nevertheless, there was a distinct difference in the results of the ice test performed on different days. Although the repeatability of the ice test (61.5%) in MG was relatively poor in our study, repeated tests may enhance the validity of the ice test.

Our study showed that repeated ice tests increased the sensitivity by 34.6% compared to a single test. The diagnostic value of the ice test in previous studies was reported to show a sensitivity of 90–95% and a specificity of 100% [3,6,12]. In contrast, the accuracy of a single ice test was much lower in our study, showing a sensitivity of 38.5% and specificity of 97.4%. Repeated ice tests increased the sensitivity to 73.1% and specificity to 97.4%. One of the reasons for this difference may be caused by the variable degree of ptosis on different days in patients with MG. That is, the degree of ptosis prior to the ice test (pre-test MRD) may be variable, as shown in our study. Previous studies have not dealt with such characteristics of MG. In cases of MG with complete ptosis, it is known that the ice test may show negative results [3]. However, even patients with complete ptosis may show variable degree of ptosis during the day [15]. In addition, the sample size of our study was larger than the previous studies, and owing to the variable degree of ptosis on different days and time of day, we speculate that this may have affected the test results, which partly explains the low sensitivity of a single ice test, and increased sensitivity with repeated tests. An additional step to induce sufficient fatigue before the ice test may be beneficial to acquire more reliable results in the diagnosis of MG. There was no statistically significant difference in the results of the ice test between ocular MG and generalized MG, unlike the anti-AChR antibody which the positive rate is much higher in generalized MG [16]. The relatively low percentage of myasthenia gravis patients in this sample who were positive for anti-AChR antibodies and repetitive nerve stimulation may probably be related to the large proportion of patients with purely ocular MG (77%) compared to patients with generalized MG (23%). Previous studies have reported that anti-AChR antibodies are positive in as many as 90% of patients who have generalized MG but in only 45–65% of those who have only ocular MG [17–20]. The decremental response in repetitive nerve stimulation was found in 70–90% of generalized MG but in only 20–35% of patients with purely ocular MG [21–24]. In contrast, there was no statistically significant difference between ocular MG and generalized MG in terms of the ice test results. Therefore the ice test may serve as the most sensitive test in diagnosing both purely ocular MG and generalized MG.

In our study, negative results were subdivided into ‘equivocal’ and ‘non-equivocal’ negative results. Among the patients with repeated negative results, 63.6% of patients who showed an equivocal negative result at least once turned out to have MG, but otherwise no patient had MG. In other words, a patient showing non-equivocal negative results repeatedly may have a very low chance of having MG. Conversely, if a patient showed an equivocal result of 1–2 mm improvement, the possibility of having MG would be 64%, and a strong suspicion of MG would be reasonable. If the ice test is judged positive when improvement of MRD is ≥ 1.0 mm at least once after repeated tests, the sensitivity is 100% (26 of 26) and specificity is 81.6% (31 of 38) (Table 4). This increases the sensitivity by 26.9%, while a decrease in specificity by 15.8% occurs compared to the original criteria. If the ice test is judged positive when improvement of MRD is ≥ 1.5 mm at least once after repeated tests, the sensitivity is 88.5% (23 of 26) and specificity is 84.2% (32 of 38). This increases the sensitivity by 15.4%, while a decrease in specificity by 13.2% occurs compared to the original criteria. Therefore, if the ice test should be performed as the initial step for diagnosing MG, equivocal negative results should be interpreted as ‘possible MG’, despite the small increase in false positive rates. Our study shows that the clinical significance of the ice test in terms of interpreting negative results, either equivocal negative or non-equivocal negative, may be important in the diagnosis of MG. In our study, cases that showed false positive results of the ice test included Miller-Fisher syndrome and ptosis secondary to internal carotid artery aneurysm. Cases showing equivocal negative results except MG included ischemic oculomotor nerve palsy with ptosis and thyroid associated ophthalmopathy. As ptosis is rare in thyroid associated ophthalmopathy and MG is more frequent in patients with thyroid disease [25,26], an equivocal negative result of the ice test may imply a ‘possible MG’ even if other test results are all negative for MG, and close observation for the symptoms of MG would be necessary.

The limitations of our study are that it was a retrospective one, and there could have been a selection bias towards patients with more variable symptoms and good compliance to repeated examinations. The sample may not be representative of the true prevalence of myasthenic blepharoptosis among patients presenting for evaluation of blepharoptosis in the general clinic. The estimated PPV/NPV reported from our study population could be different from the true value of the general population since the high prevalence of MG in our study could inflate our reported PPV/NPV. In addition, 12 patients in the MG group were taking acetylcholinesterase inhibitors prior to the ice test. However, a previous study reported that patients taking acetylcholinesterase inhibitors at the time of the ice test still had positive responses, and thus medications did not have to be stopped [3]. We verified that there was no statistically significant difference of the positive rates (58.3% vs 85.7%) and equivocal negative rates (41.7% vs 14.3%) of the ice test between MG patients taking anti-cholinesterase inhibitors before the test and those who did not (*p* = 0.116, χ^2^ test). Generally, determining repeatability requires multiple measures to examine the correlation between each measure in a regression model which allows for additional levels of adjustment. However, there are certain limitations in examining the test multiple times due to potential ethical issues, cost-effectiveness and patient-inconvenience. Further well controlled researches to investigate the factors causing different results of the ice test may be helpful, particularly the degree of ptosis before the test. It would also be beneficial to ascertain the usefulness of inducing fatigue as an additional step before the ice test.

## Conclusions

The repeatability of the ice test was 61.5% in myasthenic ptosis. Repeated tests enhanced the sensitivity of the test by 34.6% compared to a single test. Equivocal negative results should be interpreted with caution, as these patients have a possibility to have MG in 63.6%.

## Supporting information

S1 File Minimum dataset file with supporting information. The minimum dataset file including demographics and test results of patients with ptosis related to myasthenia gravis and controls.
(XLSX)
